# Once again... breakthrough cancer pain: an updated overview

**DOI:** 10.1186/s44158-023-00101-x

**Published:** 2023-07-21

**Authors:** Sebastiano Mercadante

**Affiliations:** Anesthetics, Main Regional Center for Pain Relief & Palliative Care Unit, La Maddalena Cancer Center, Acute Supportive/Palliative Care Unit & Hospice, 90146 Palermo, Italy

**Keywords:** Cancer pain, Breakthrough pain, Opioids, Fentanyl, Morphine, Palliative care

## Abstract

Breakthrough cancer pain (BTcP) is a complex and variegate phenomenon that may change its presentation during the course of patients’ disease in the same individual. An appropriate assessment is fundamental for depicting the pattern of BTcP. This information is determinant for a personalized management of BTcP. The use of opioids as needed is recommended for the management of BTcP. There are several options which should be chosen according to the individual pattern of BTcP. In general, a drug with a short onset and offset should be preferred. Although oral opioids may still have specific indications, fentanyl products have been found to be more rapid and effective. The most controversial point regards the opioid dose to be used. The presence of opioid tolerance suggests to use a dose proportional to the dose used for background analgesia. In contrast, regulatory studies have suggested to use the minimal available dose to be titrated until the effective dose. Further large studies should definitely settle this never ended question.

## Key points

- Breakthrough cancer pain is a complex and variegate phenomenon.
- An appropriate assessment is fundamental for defining the characteristics.
- Opioids still remain the pharmacological option for treating breakthrough pain.
- Management is based on the individual characteristics of breakthrough pain.
- The choice of opioid doses should be based on the level of tolerance.

## Background

In the last 30 years, physicians have paid more and more attention to the phenomenon of breakthrough cancer pain (BTcP) [1]. Clinicians dealing with cancer pain become aware that peaks of pain overlapping persistent pain require a different evaluation and treatment. In the past decades, fluctuations in persistent pain were afforded increasing opioid doses prescribed for background pain, as if it was a condition of global uncontrolled pain, often resulting in oversedation when patients were out of any episode or at rest. The presence of BTcP is deemed to have a negative impact on general activities, quality of life, and pain management [2, 3].

In the 1990s, the definition of BTcP focused on some characteristics to better characterize this phenomenon, providing more insights on this phenomenon [4–9]. The reported prevalence is dependent on the population studied and ranges from approximately 40 to 80%, depending on the definitional criteria [1]. With a lowered cutoff for background pain intensity, the reported prevalence seems to decrease [10].

Studies published in the last 10 years confirmed that BTcP is a variegate phenomenon, with different presentations that may change during the course of patients’ disease [11, 12]. In addition, many patients may experience more types of BTcP [13]. Rather than defining BTcP as a phenomenon with a typical pattern, it is likely that the plural term of “breakthrough pains” is more adequate.

This narrative review will explore some aspects regarding the assessment and the management of BTcP to give an updated overview of this phenomenon.

## Assessment

To depict the pattern of BTcP in individuals and to provide accordingly a specific treatment, an appropriate assessment is of paramount importance. Thus, a proper definition should be given a priori. BTcP has been defined as transitory increase in pain to greater than moderate intensity, which occurs on a baseline pain of moderate intensity or less. This definition resulted to be a little bit ambiguous in literature, as many studies included different patterns of patients, for example, receiving or not opioids, having or not controlled background pain, and experiencing severe or less severe episodes of BTcP. In literature, studies of BTcP often included patients with mild-moderate pain having BTcP episodes of moderate-severe pain, resulting in a gray area which included a relatively high background pain intensity and a low BTcP intensity [14–18]. The inclusion of such patients posed serious biases on the evaluation of data, particularly when comparing BTcP medications with placebo, possibly increasing the placebo effect [19]. Thus, the level of pain intensity which is considered acceptable for patients could be important to provide a common parameter, as it expresses a relevant individual measure to be considered in this context [20]. In recent years, there was an agreement to identify BTcP.

First, to define BTcP, patients must be receiving a stable and effective analgesic regimen, providing well-controlled background pain for most hours of the day. Indeed, the peak of BTcP should be moderate-severe in intensity and should be clearly distinguishable from the background pain intensity. It has been reported that at least 3 points of differences on the numerical pain scale give the input to request a medication [20].

Interestingly, patients receiving lower doses of opioids with an adequate background analgesia develop episodes of BTcP with a prevalence similar to that reported in general population of cancer patients. This group of patients was on an early stage of disease and had less aggressive pain syndromes and less interference with daily activity, probably due to a lower number of episodes with a lower intensity. It is likely that for this reason, this subgroup of patients was less frequently prescribed a BTcP medication. Of interest, patients showed a longer time to meaningful pain relief and were less satisfied with BTcP medications which were mainly oral morphine [21]. This aspect has consequent clinical implications, suggesting the need of different strategies.

Secondly, the temporal pattern should be taken into consideration. The onset of BTcP is relatively short, reaching a peak within 10 min in about 2/3 of cases. The offset is variable, 45 min on average, ranging from minutes to 1–2 h. Most of these episodes vanish spontaneously [7, 8]. The persistence of untreated pain after 1–2 h suggests that background pain is not well controlled and needs an opioid dose adjustment. On the basis of these consideration, BTcP should be considered as an episode of severe pain of short onset and offset, which occurs in patients who are receiving a stable analgesic regimen, stable baseline pain mild in intensity, and clinically acceptable.

Third, BTcP may be spontaneous or incident, due to a precipitating event. *Spontaneous or idiopathic BTcP lacks an recognizable cause or a precipitating event. These peaks of pain intensity are characterized by a gradual onset, prolonged duration, and slow offset*. In the incident-type BTcP, pain is triggered by an identifiable factor that can be predictable or not. In contrast, this type of BTcP often has a rapid onset and short duration. The most common types of incident BTcP are movement-induced bone pain, frequently due to bone disease, and swallow-induced pain, due to oral mucositis [7, 8]. In advanced cancer patients, BTcP presentation may be variegate, with concomitant forms of BTcP with different mechanisms, including both predictable and unpredictable episodes [13].

Fourth, the number of episodes per day in another element is to be considered. The occurrence of 3–4 BTcP episodes per day is commonly considered acceptable when pain is controlled during the rest of the day [5, 6]. In these circumstances, the balance between drugs given around the clock and medications given as needed may be maintained. However, in some clinical circumstances, this statement may appear as generalization. For example, predictable episodes of incident pain due to movement in patients with bone metastases depend on the level of patient’s activity. It may subside if the physical activity is stopped [7, 12]. Alternately, optimization of background opioid therapy may improve mobilization [22]. In other words, the need to use a BTcP medication may not be determined by an arbitrary number of painful episodes. Rather, it depends on the balance between activity and background analgesia and the reliability of a BTcP medication by the patient’s individual preference. Some subjects prefer to carry out some activities and tolerate a large number of BTcP episodes each day, while some others use to restrict their activity to prevent BTcP episodes. This frequently occurs in bedridden patients with a low level of activity, for example, fractured patients [1]. Patient preference will be the key on treatment decisions. From a therapeutic perspective, the aim should be attempting to optimize the background analgesia based on a tailored use of opioids, eventually supported by the use of adjuvant analgesics to find the best compromise between background analgesia and desired activity.

Fifth, pain mechanisms should be considered. For example, some forms of neuropathic pain may produce BTcP episodes with an onset and an offset of few seconds or minutes. In this case, no drug will have such temporal pattern. In this case, BTcP events should be prevented with the preemptive use of adjuvant drugs, rather than treating the single BTcP event [1].

Sixth, some tumors may have some peculiarities. Patients with head and neck cancer report a higher number of episodes of BTcP, which were predictable, particularly with the ingestion of food, which was the main precipitating event. This may be explained by the prevalence of mucositis of severe intensity, inducing pain on swallowing [23]. Similarly, patients with pancreatic pain often report postprandial pain [24]. It goes without saying that different strategies can be used based on preemptive analgesia rather when the episode occurs.

Seventh, BTcP is often a sentinel event of a pain that is not well controlled as previous analgesic treatment loses efficacy, meaning that BTcP is related to insufficient opioid therapy. The development of more frequent episodes of BTcP is a marker of poor analgesia. This distinction is fundamental, because the relative intervention is much different (see below).

Finally, the characteristics of BTcP may change during the course of disease. Different studies have shown that advanced cancer patients having a lower Karnofsky level and followed by a palliative care team, and possibly severely ill, experience a lower number of BTcP episodes/day, with a slower onset, and less predictability than patients evaluated in a pain clinic or oncologic ward, possibly visited in an early phase of disease [11, 12, 25]. Thus, during the progression of disease, the pattern of BTP may change, so influencing the goals of care [26].

The knowledge of the different types of BTcP may allow an individualized care planning and a global better outcome. Pain characteristics, optimization of background pain, disease status, and patient preference should be the basis for the treatment of BTcP, which is likely to change over time.

## Strategies for the pharmacologic treatment of BTcP

Although different non-pharmacological methods have been proposed, the evidence for efficacy is very poor [27]. The use of opioids as needed remains largely recommended for the management of BTcP. Large studies have shown that in most BTcP episodes, the peak in pain intensity develops within a few minutes and lasts for 30–60 min [7, 8]. For these reasons, a drug with a short onset and offset should be selected to avoid the effect of the drug which is protracted when most episodes spontaneously disappear. It is likely that such lasting effect may produce adverse effects, once the level of pain is under control, as it was before the event.

### How to choose opioids for BTcP

*Varying recommendations from different organizations have been published. Current guidelines agree on many aspects of the management of BTcP. However, the evidence regarding such guidelines remains low grade *[28]*. For example, the European Association for Palliative Care (EAPC) guidelines suggest oral opioids as the first-line treatment, with the use of ROOs recommended only for episodes with rapid onset and shorter duration of effect *[28]*. The National Institute for Clinical Excellence (NICE) guidelines suggested that morphine should be considered the first-choice drug for BTcP *[29]*. Indeed, the European Society for Medical Oncology (ESMO) recommends the use of ROOs as a first-line treatment *[28]*.*

Oral opioids have been used for years, possibly because there were no alternatives for a rapid analgesic effect, unless the injectable formulations are given parenterally. From a pharmacological point of view, there is a mismatch between the time course of a BTcP episode and the typical time-action relationship of oral opioids. Oral opioids have a slow onset of effect, ranging 30–45 min, providing a late initial analgesia when the majority of BTcP episodes are resolved spontaneously. In addition, the duration of effect is much longer than the typical BTcP temporal wave. It is likely that an eventual benefit reported after taking an oral opioid for BTcP is expectational, rather than pharmacological [1]. On the other hand, patients may prefer a “single-shot drug,” not engaging unfamiliar delivery systems. *NICE recommendations were mainly based on an economic criterion rather than on effectiveness, reflecting concerns about the higher cost of fentanyl preparations. In contrast to the suggestion of prescribing oral morphine as first choice for treating breakthrough pain episodes*, a recent double blind randomized study confirmed the inadequacy of oral morphine. Different doses of oral morphine were compared with placebo were ineffective for relieving BTcP, and some pain relief was observed only after about 2 h, just when any “typical” BTcP event spontaneously evanishes [30]. Nevertheless, oral opioids may still have indications for treating some forms of BTcP. For example, the predictability of BTcP should be considered in characterizing acceptable outcomes. Preemptive analgesics could be given before the pain occurs. Oral morphine could be given 30 min before starting an activity expected to induce pain with a gradual onset and lasting some hours. Such preemptive analgesia for a predictable BTcP, however, requires a high level of patient education about the right timing of drug administration in relation to the onset and duration of the pain [1]. This tailored approach can be effective for patients who are capable to manage the use of preemptive drugs on their own. Similarly, postprandial BTcP, which has a slow onset and long duration, could be eventually prevented by a sort of appetizer, giving a preemptive oral morphine half an hour before food ingestion. Education on the proper timing for administration will be determinant on the efficacy of this approach. In patients with postprandial BTcP, the prophylactic use of oral opioids was effective and did not add risks of toxicity [31]. This strategy has obvious implications on the nutritional status and quality of life, because patients may avoid to eat to prevent the development of BTcP. In clinical practice, the factors that influenced the pharmacological treatment for BTcP were background opioid dosage, setting of assistance, and selfability to take medication [32].

Various transmucosal fentanyl formulations provide a fast analgesic effect, within 5–10 min. All controlled studies of transmucosal fentanyl preparations demonstrated superiority over oral opioids and placebo, with a time-action relationship supporting a faster onset than oral opioids and yielded more favorable outcomes [33, 34].

Transmucosal fentanyl provides fast analgesia as it rapidly pass the mucosa and then the blood–brain barrier due to its potency and lipophilicity. Transmucosal fentanyl preparations belong to n heterogeneous group of delivery systems. The availability ranges from 50% (oral transmucosal fentanyl citrate, OTFC) to 90% (intranasal fentanyl). Fentanyl pectin nasal spray (FPNS), buccal fentanyl (FBT), and sublingual fentanyl (SLF) have an availability of 60–65%. Each formulation has its peculiarity and could be chosen according to the clinical condition of nasal and oral cavities, ease of use, and patient’s preference.

These fentanyl formulations all have been tested in opioid-tolerant patients receiving at least daily doses of 60 mg or greater of oral morphine equivalents (OME). This approach was proposed to improve safety and prevent any risk of respiratory depression.

On the other hand, some patients may receive doses of OME for background pain lower than 60 mg/day of OME. For these reasons, oral morphine was more frequently used, likely due to prescription requirements for patients with relatively low opioid exposure [21], resulting in unsatisfactory pain relief, longer time to meaningful pain relief, compared to patients receiving higher doses of OME who were receiving fentanyl products. Although prescription regulations require that the minimal strength of fentanyl preparations should be given in patients receiving at least 60 mg of OME for the risk of adverse effects [35], the percentage of patients reporting adverse effects was lower compared to that found in the group of patients receiving more than 60 mg of OME. This is consistent with the finding that the lowest strength of transmucosal fentanyl can be tolerated even by patients receiving less than the traditional 60 mg/day of OME. For instance, a dose of 67 µg of fentanyl was effective and safe in this category of patients [36].

### How to dose opioids for BTcP

Another controversial point regards the choice of the dose of fentanyl products to be prescribed for BTcP episodes. Early clinical trials of the transmucosal fentanyl formulations provided data for regulatory approval and safety issues. These studies used a paradigm of a dose-finding approach, suggesting to start with the lowest existing dose of the formulation and then to increase until the effective dose was reached. This statement, however, was passed off as evidence based, because the subsequent part of the study design, which is the comparison of the successful dose with oral opioids or placebo, was double blind and controlled according to the best evidence studies. However, the part of dose finding was open label, resulting in an enrichment study recruiting only patients responsive to dose-finding protocol, and excluding patients who did not respond or not achieved the effective dose. According to this data, no relationship between the effective transmucosal dose and the daily dose of the opioid taken for baseline pain was found in a secondary analysis. Thus, the approval of the formulations incorporated instructions was incorporated to approve the formulations and give instructions to start therapy at the lowest doses available for each product and then titrate to the effective dose [37].

This approach, however, is pharmacologically contrary to the dose–effect proportionality typical of opioid drugs. Although dose titration increases early safety overall, it could result to be problematic for some patients, resulting in prolonged periods of unsuccessful treated events [37, 38]. The use of repeated doses of fentanyl products could lead to prolonged drug exposure and can be frustrating, increasing the uncertainty and discouraging patients to use BTcP medications, and also reducing their acceptability and patients’ compliance [39].

There are other aspects deserving a deeper evaluation of early randomized trials regarding the finding of a lack of association between the fentanyl dose and the opioid dose used for background pain. In these studies, patient selection was not based on modern criteria of BTcP definition, as levels of background pain were unexpectedly high while BTcP intensity was not too high. This observation explains the amplification of the placebo effect [37, 38]. Even not well-numbered, a comparative study showed that FBT in doses proportional to the baseline opioid dose was more effective than titrated doses of FBT, with comparable risks of adverse effects [40]. Dose proportionality is pharmacologically explainable, based on the concept of opioid tolerance, for which in a patient with a peak of pain intensity one needs to increase the opioid plasma concentration to produce a larger effect in a patients receiving a certain dose of opioids and one needs to give a dose of such an amount as to produce a greater effect.

The proportional approach has been assessed in several studies, also including patients receiving high doses of opioids and older patients [41–46].

In comparison studies, the use of FBT and FPNS in doses proportional to those used for background analgesia was substantially superior over oral morphine during the first 30 min of administration [44, 45].

In the real world, the prescription of opioid dose used for BTcP was based on the proportionality [47, 48]. A study confirmed the efficacy and safety of fentanyl in doses proportional to the baseline opioid regimen [49]. This line of thinking, based on the level of tolerance, is not necessarily associated with more risks just because the level of tolerance has a protective role against the risk of respiratory depression.

Indeed, when taking into consideration the dose-finding and proportional methods, there is a need of a greater flexibility when prescribing opioid for BTcP. Low doses of transmucosal fentanyl could not be optimal in an opioid-tolerant patient receiving high opioid doses for their background analgesia. As these patients are highly opioid tolerant, the titration process would be time-consuming and frustrating. *Thus, patients with distressing BTcP episodes, who are highly tolerant to opioids and do not have serious comorbidities or frailty, should be given a dose roughly proportionate to the dose used for background pain*. A possible compromise between the two different methods should be skipping some steps of dose titration for patients receiving high doses of opioids for background analgesia.

## Conclusion

BTcP is a variegate phenomenon which requires a careful assessment for the different clinical presentations, which are variable in individuals and often changing in the same individual. The clinical pattern will help in finding the most appropriate management. A personalized approach is of paramount importance when prescribing a medication for BTcP, and careful attention should be given to drug choice, doses, and route of administration, considering both pain- and patient-related factors. Further studies should investigate on alternative treatments, *comparing the different available substances in different conditions and providing more solid data regarding dosing*.

## Data Availability

NA.
